# Evaluating pain outcomes in Chinese ophthalmology patients using the APS-POQ-R-C: a Rasch analysis

**DOI:** 10.3389/fpsyg.2025.1558111

**Published:** 2025-05-05

**Authors:** Meina Huang, Zheyi Chen

**Affiliations:** ^1^Department of Surgery, Eye Hospital and School of Ophthalmology and Optometry, Wenzhou Medical University, Wenzhou, Zhejiang, China; ^2^National Clinical Research Center for Ocular Diseases, Wenzhou, Zhejiang, China; ^3^Department of Optometry, Eye Hospital and School of Ophthalmology and Optometry, Wenzhou Medical University, Wenzhou, Zhejiang, China

**Keywords:** pain management, APS-POQ-R-C scale, Rasch analysis, ophthalmic surgery, postoperative pain

## Abstract

**Background:**

This study aimed to evaluate the psychometric properties of the Chinese version of the Revised American Pain Society Patient Outcome Questionnaire (APS-POQ-R-C) using Rasch analysis, to optimize the APS-POQ-R-C for effective pain assessment in Chinese postoperative ophthalmic patients.

**Methods:**

The polytomous analysis approach of the Rasch model was used to comprehensively evaluate the applicability of the APS-POQ-R-C scale in postoperative ophthalmic patients. Using a sample of 294 valid questionnaires, multiple aspects of the scale were tested, including unidimensionality, local independence of items, reliability and separation, item fit, person–item mapping, test information function, and differential item functioning (DIF) analysis.

**Results:**

Principal component analysis of residuals, explained common variance (0.61) and omega hierarchical (0.72) of the APS-POQ-R-C scale demonstrates essential unidimensionality. The reliability and separation of person were 0.93 and 3.64, item were 0.99 and 10.32, indicating high reliability and separation. The standardized residual correlations between items were all below 0.7, suggesting local independence. The response category functioning results recommended merging categories 8, 9, and 10. Except for item P10, most items had infit and outfit mean square (MNSQ) values within acceptable ranges, indicating good fit to the Rasch model. Item P10’s MNSQ values exceeded 1.50. The person-item map indicating that item difficulty was generally higher than the mean ability of the population. The test information curve showed that the scale was most informative for individuals with higher levels of the latent traits. DIF analysis revealed slight gender-related differential functioning in items P5a, P5b, P5c, and P5d, with absolute DIF contrast greater than 0.5.

**Conclusion:**

The APS-POQ-R-C can be used to assess postoperative pain management effectively in the study sample, with overall good psychometric properties. Further optimization is suggested, including reducing item redundancy, incorporating more simple items and considering the potential influence of gender differences on responses to the scale.

## Background

Pain is one of the most common symptoms in human patients, particularly in postoperative or cancer patients (Kwon et al., 2013). Research has revealed that up to 75% of patients experience postoperative pain, with as many as 30% suffering from moderate to severe postoperative pain (Awan and Durrani, 2015). Unrelieved pain often leads to severe physical and psychological impairments, negatively impacting patients’ disease prognosis and quality of life (Manjiani et al., 2014). Consequently, healthcare professionals are increasingly prioritizing pain elimination or, at the very least, reduction to a tolerable level, with pain management playing a crucial role (Zoega et al., 2014). Proactive and effective pain management can improve patient prognosis, prevent postoperative complications, reduce healthcare costs, and ultimately enhance patient quality of life (Apfelbaum et al., 2003).

Postoperative pain during ophthalmic surgery is a relatively understudied area of clinical research. This is due to the limited trauma caused by ophthalmic surgery compared to other surgical procedures, resulting in seemingly milder postoperative pain (Coppens et al., 2002). However, studies have demonstrated that ophthalmic surgery can also lead to severe postoperative pain. Moreover, in ophthalmic surgery, short-acting anesthesia is often employed to facilitate patients’ rapid recovery from anesthesia, rendering them more susceptible to early postoperative pain. Research has shown that the incidence of severe postoperative pain in elective ophthalmic surgery ranges from 13.8% in procedures such as cataract surgery and iridectomy to 53.8% in strabismus surgery (Henzler et al., 2004).

Effective pain management necessitates a scientific and systematic pain assessment as its foundation. Pain is an inherently subjective phenomenon characterized by its multidimensional nature (Hjermstad et al., 2011). Effective pain management must be based on valid pain assessment questionnaires (Gordon et al., 2005). In the Chinese population, several pain assessment tools have been validated, such as the McGill Pain Questionnaire (MPQ), which is widely used to assess pain intensity, quality, and sensory and emotional components. However, these tools, including the MPQ, may not fully capture the complexity of pain experiences specific to Chinese postoperative patients, particularly in the context of ophthalmic surgery (Wang J. L. et al., 2017; Tsui et al., 2024). A comprehensive pain assessment should encompass not only pain intensity but also considerations of pain duration, medical interventions, and various other contributing factors (Gordon et al., 2010). The most widely used postoperative pain assessment questionnaire in clinical practice is the American Pain Society Patient Outcome Questionnaire (APS-POQ), which was established in Bond et al. (1991) and underwent revisions in 2005 and 2010, resulting in the APS-POQ-R (Gordon et al., 2005; Gordon et al., 2010). The APS-POQ-R assesses multiple dimensions, including pain intensity, impact on function and emotions, patient satisfaction, and more, and is considered to comprehensively reflect pain management quality (Zoega et al., 2014; Gordon et al., 2010). A critical comparison of the APS-POQ-R with other validated pain assessment tools, such as the MPQ, reveals notable differences in their focus and structure. The MPQ is well-known for its comprehensive evaluation of pain quality and its ability to capture a wide range of sensory and affective pain dimensions. However, the APS-POQ-R provides more direct insights into the broader context of postoperative care, including treatment side effects, patient involvement in pain management decisions, and satisfaction with care. Unlike the MPQ, the APS-POQ-R is specifically tailored for assessing pain management outcomes in postoperative settings, making it particularly relevant for evaluating ophthalmic surgery patients in China. The APS-POQ-R was translated into 11 languages (Dihle et al., 2006; Dihle et al., 2008). Gordon et al. (2010) used the English version of the APS-POQ-R to assess postoperative pain management in 299 patients and reported that the APS-POQ-R has good internal consistency and structural validity. Zoega et al. (2014) used the Icelandic version of the APS-POQ-R to assess postoperative pain management in 143 patients and found that the questionnaire also exhibited good reliability and structural validity. However, these studies were based on Caucasian populations. Numerous studies have highlighted that pain sensitivity and tolerance differ among ethnic groups. For instance, Holmgaard et al. (2017) reported that individuals with dark eyes and hair exhibit greater pain sensitivity. Therefore, it is essential to investigate the application of the APS-POQ-R in Asian populations. Fang et al. (2017), Wang H. et al. (2017), and Wang et al. (2021) used the Chinese version of the Revised American Pain Society Patient Outcome Questionnaire (APS-POQ-R-C) to assess postoperative pain management in Chinese patients and confirmed its good reliability and validity for measuring postoperative pain in the Chinese population.

However, the aforementioned assessments of the questionnaire employed classical test theory, which, despite its widespread use, has limitations. Its dependence on the sample and the questionnaire renders it impossible to compare respondents across different samples or different versions of the questionnaire, causing inconvenience in the scale’s application. The analysis results based on classical psychometric theory are easily influenced by the sample’s ability and the content of the questionnaire, implying that administering the same questionnaire to different groups of subjects will yield different results (Embretson et al., 2000). Furthermore, classical measurement theory assumes that the raw scores of the questionnaire are equidistant, which is inconsistent with reality.

To address the limitations of classical theory, item response theory (IRT) has emerged (Lord, 1952). This theory postulates that the response to each item in the measurement depends on the respondent’s level of a certain trait. Therefore, item response theory is frequently denoted as latent trait theory or the latent trait model. Item response theory assumes that subjects possess a latent trait, which is a statistical hypothesis proposed based on the observation and analysis of measurement responses. In measurement, latent traits generally refer to latent abilities, and the total measurement score is often used as an estimate of this potential. Item response theory posits that subjects’ responses and results on measurement items have a unique relationship with their latent traits. This theory establishes scale item parameters with the characteristic of permanence, allowing the unification of scale scores across different respondents. The Rasch model (Rasch, 1960) constructs an objective interval scale through strict unidimensionality and parameter invariance, making it the gold standard for scale optimization, enabling precise single-dimensional assessments. Using the Rasch model to analyze scales offers the following advantages: (1) item parameter estimates independent of the sample can be obtained; (2) whether different populations have response biases can be tested; (3) whether the threshold order of each category item is correct can be assessed; and (4) whether items exhibit local dependence can be evaluated. However, when research involves multidimensional interactions, its limitations emerge. Multidimensional IRT models (Embretson et al., 1986) address this by introducing multiple latent variables and discrimination parameters, allowing items to span dimensions and reflect complex cognitive mechanisms. The Rasch model ensures theoretical purity for unidimensional validation, while multidimensional IRT models offer flexibility for complex analyses.

Rasch analysis is widely applied in China to assess psychometric properties of scales, particularly in ocular pain and surgery outcomes. The Ocular Pain Assessment Survey (OPAS) has been validated for Chinese populations with dry eye and neuropathic corneal pain (Ng et al., 2025), revealing that some dimensions need refinement. Similarly, the 5-Dimension Comprehensive Assessment Scale (5DCAS) was validated for assessing physical function in Chinese patients with axial spondyloarthritis, showing good reliability (Zheng et al., 2024). The Hong Kong Quality of Life Questionnaire (HKQ), re-engineered for cataract surgery outcomes, also underwent Rasch analysis to improve its precision for Chinese patients (Khadka et al., 2018). These studies emphasize the value of Rasch analysis in refining outcome measures.

Therefore, using Rasch model to analyze the APS-POQ-R-C is highly important. First, parameters such as item difficulty and discrimination can be obtained, providing a basis for scale optimization. Second, it can test whether different populations (such as patients of different ages and gender) have consistent responses. Third, it can assess whether the category item functions normally. Finally, this study provides a foundation for future clinical applications. However, to date, no relevant research reports have been published. Consequently, this study aimed to use Rasch model to analyze the characteristics of the APS-POQ-R-C scale in postoperative ophthalmic patients, with the goal of providing a basis for its optimization and better application in clinical practice.

In summary, the standardization of postoperative pain management in ophthalmology urgently requires the use of scientific assessment tools as a foundation. As a relatively comprehensive multidimensional pain outcome scale, the APS-POQ-R-C lacks Rasch model analysis. This study will conduct a detailed and comprehensive Rasch examination of the scale, provide suggestions for scale optimization, and support improvements in postoperative pain management in ophthalmology.

## Methods

### Ethical consideration and subjects

This study was approved by the local ethics committee (No. 2023-157-K-129-01) in accordance with the Declaration of Helsinki (2013) and conducted at the Eye Hospital of Wenzhou Medical University, with participants being adult ophthalmic patients undergoing surgery at the hospital’s surgical center. Participants were adult patients recruited between November 2023 and April 2024 who underwent various ophthalmic surgeries (e.g., cataract, corneal, vitreoretinal, glaucoma, strabismus, and ocular tumor surgeries). Inclusion criteria: Chinese adults > 18 years, native Mandarin speakers, postoperative hospital stay > 24 h, and informed consent. Exclusion: Cognitive or mental disorders.

### Instrument

The APS-POQ-R-C was adapted for use in the Chinese population to provide a culturally relevant tool for evaluating postoperative pain experiences (Fang et al., 2017; Wang H. et al., 2017; Wang et al., 2021). This version maintains the multidimensional structure of the original APS-POQ-R, encompassing additional factors such as side effects of pain treatment, patient participation in management decisions, and pain relief efficacy, while also being tailored to the specific needs of Chinese patients. The sample size was determined based on the general recommendation to enroll 10 to 20 participants per item in the questionnaire. In this study, a total of 294 participants were enrolled, which falls within this recommended range. Patients were asked to read and sign the consent form before completing the questionnaire independently.

### Item response theory analysis

To evaluate the psychometric properties of the APS-POQ-R-C scale using Item Response Theory (IRT), we employed the Rasch model, which assumes essential unidimensionality. Therefore, a thorough assessment of the scale’s dimensionality was conducted prior to fitting the final IRT model. Dimensionality assessment involved two primary methods.

#### Principal component analysis of residuals

This was performed using Winsteps software (version 3.74.0, Illinois, United States) on the residuals obtained from an initial Rasch analysis of all scale items. We examined the structure of the residuals, particularly the eigenvalue of the first contrast. The analysis confirmed if the first principal component accounted for more than 50% of the total variance, supporting the unidimensionality of the scale, eigenvalue of the first contrast less than 3.0 was considered as additional evidence supporting the unidimensionality (Linacre, 2013). Varimax rotation was applied to further clarify the factor structure and ensure that the items loaded appropriately onto the first principal component.

#### Omega hierarchical and explained common variance

The two indexes were calculated using the omega function by R-4.4.3 (CRAN, United States) with psych package. The explained common variance (ECV) index is considered the best measure of the degree of unidimensionality (Reise et al., 2013). It provides information on the proportion of the common variance attributable to the general factor. An ECV value above 0.60 indicates, at least, substantial one-dimensional nature of the tool, ωh estimates the proportion of reliable variance in the total score that can be attributed to a single general factor, accounting for potential sub-dimensions (Liu et al., 2023).

Local independence was verified by examining standardized residual correlations between items using Yen’s Q3 (Yen, 1984), with values above 0.70 suggesting redundancy (Linacre, 2013; Linacre, 2018). Reliability and separation indices for both persons and items were calculated, with values above 0.80 (Crocker and Algina, 1986) for reliability and greater than 2 (Duncan et al., 2003) for separation considered optimal. These metrics demonstrated the scale’s ability to consistently rank individuals and items while distinguishing between different levels of pain intensity. Item characteristics were assessed for misfitting items, with problematic items flagged for revision or removal. Fit statistics (outfit and infit MNSQ) were within the ideal range of 0.5–1.5 (Linacre, 2002; Wright et al., 1994; Nunnally and Bernstein, 1994); values outside this range indicated potential misfit. Person-item maps (Wright maps) (Boone and Noltemeyer, 2017) were used to visualize the alignment between person ability and item difficulty. Test information curves were analyzed to ensure the scale provided adequate precision across the full range of pain levels (Baker, 2001). Finally, DIF analysis tested for age and gender related bias in item responses (Linacre, 2013). A DIF contrast value above 0.5 indicated mild bias, and values exceeding 1.0 suggested the need for item modifications. For age grouping, individuals were classified as “older” if aged > 64.23 years (mean age), and “younger” if aged < 64.23 years.

### Questionnaire collection and data processing

A nurse, not involved in patient treatment, collected the questionnaires, explained the study’s objectives, and, when necessary, verbally administered items to accommodate literacy limitations. Patient identities were not disclosed, and demographic data (age, gender, surgery type, pain management details) were sourced from medical records without identifiers. Each questionnaire was assigned a unique numeric identifier.

To assess the psychometric properties using Rasch analysis, we first screened items. Items P11, P12, and P13 were excluded as they were weakly related to the core construct. P11 and P12 focused on nonpharmacological treatments, while P13 concerned assistance with completing the questionnaire, all of which did not directly reflect postoperative pain management outcomes. Items P7, P8, P9, and P10, which had opposite scoring directions, were reverse scored (i.e., 10-a, 10-b, etc.) before analysis to maintain consistency in the scale direction.

The exclusion of participants with incomplete data was done to maintain the integrity of the statistical analysis, as missing data could bias the results and affect the validity of the psychometric evaluations. Only complete responses were included in the final dataset to ensure that each participant’s responses accurately reflected their pain management experience, without the influence of missing values.

### Data quality control measures

To ensure the reliability of the data, we implemented several quality control measures: Careless Responders: In addition to person fit statistics, participants with invariant responses (e.g., answering all items in the same response) were flagged and excluded if suspicious patterns were observed.

Univariate Outliers: Univariate outliers were initially flagged for each item using the interquartile range (IQR) method (Tukey, 1977). Specifically, responses falling below Q1 − 1.5* IQR or above Q3 + 1.5*IQR were flagged. These flagged values, along with visual inspection of item distributions using histograms and boxplots, were used to identify potential outliers. Outliers were only excluded if further inspection confirmed them to be data entry errors or clearly nonsensical responses.

Multivariate Outliers: Mahalanobis distance (D^2^) was calculated across 19 items to identify potential outliers. The critical threshold was set using the chi-square (χ^2^) distribution with df = 19, with a *p* < 0.001 criterion, yielding a critical value of 43.82. Cases with D^2^ > 43.82 were flagged as potential outliers and reviewed individually. Only confirmed data errors were removed from the dataset before further analysis (Ghorbani, 2019).

### Statistical methods

This was a cross-sectional study. Statistical analysis was performed using SPSS software (version 26.0, IBM Corporation, United States) and R-4.4.3 (CRAN, United States) with psych package. Rasch analysis was conducted using Winsteps software (version 3.74.0, Illinoi, United States). The measurement data are expressed as the Means ± SD (standard deviations), while the count data are expressed as numbers and percentages. A *p* value < 0.05 was considered to indicate statistical significance.

## Results

### Description of the participants

A total of 321 questionnaires were collected from postoperative ophthalmic patients. However, to ensure the accuracy and integrity of the analysis, participants with incomplete questionnaire data were excluded from the analysis. Specifically, 14 participants were excluded due to missing data, 13 participants were excluded due to low quality data, resulting in a final sample size of 294 participants for the Rasch analysis., including from 160 females (54.42%) and 134 males (45.58%), with a mean age of 64.23 ± 11.41 years (range: 39–87).

### Dimensionality assessment

The assessment of essential unidimensionality for the APS-POQ-R-C scale yielded the following results. (1) Principal component analysis of residuals (PCAR): The analysis of standardized residuals from the initial Rasch model in Winsteps revealed structure beyond the primary Rasch dimension. The first principal component explained 58.1% of the variance, supporting the unidimensionality of the scale, however, the eigenvalue of the first contrast was 5.2, which is greater than the commonly cited threshold of 3.0, suggesting the presence of secondary dimensions or systematic variance in the residuals after accounting for the main trait. (2) Explained common variance (ECV): Despite the notable first contrast eigenvalue, the ECV calculated in R was 0.61. This value exceeds the recommended benchmark of 0.60, indicating that the primary Rasch dimension accounted for over half of the common variance among the items. (3) Omega hierarchical (ωh): The calculation using the psych package in R resulted in an ωh value of 0.72. This suggests that approximately 72% of the reliable variance in the APS-POQ-R-C total score can be attributed to a single general factor. While the PCAR eigenvalue indicated potential multidimensionality, the ECV value (0.61) met the threshold, and more importantly, the ωh value (0.72) provided substantial evidence that a dominant general factor underlies the scale items. Integrating these findings, we concluded that the APS-POQ-R-C scale demonstrates essential unidimensionality to justify the application of a unidimensional Rasch model to all items for subsequent psychometric analysis.

### Reliability and separation

Testing of the APSPOQ-R-C showed strong reliability and separation for both items and persons. High person reliability (0.93) and separation (3.64) suggest the scale effectively distinguishes between individuals at different levels, while high item reliability (0.99) and separation (10.32) indicate the items accurately reflect latent traits.

### Local independence

None of the items beyond the 0.7 limit indicating item independence in the scale (Linacre, 2002). Almost all the residual correlation values were between −0.30 and + 0.30, the small absolute correlation values (below 0.4 for most) suggest minimal overlap, supporting low dependence. The negative correlations align with the scale’s structure, where items reflecting different traits should be negatively correlated. Only 8 of 171 pairs showed Q3 between 0.4 and 0.53 due to item content similarity. For instance, P5a-P5b showed Q3 value of 0.53, P5b-P5d (0.52), P4b-p4d (0.50). Given these values do not exceed the threshold for significant local dependency (0.7), and align with theoretical expectations (e.g., anxiety-depression comorbidity in mental health scales), although they may be higher than expected and may indicate some degree of relatedness between these items, considering clinically meaningful distinctions, we recommend retaining all items in this analysis.

### Monotonicity

The Andrich thresholds for the three dimensions showed a consistent increase from negative to positive values, with no abnormalities. Category measures steadily increased with higher ratings, and the outfit MNSQs for all categories were under 2, indicating good model fit. Observed and expected values were consistent, with no significant differences. Overall, the rating category structure demonstrated good monotonicity, supporting accurate scale measurement.

### Category probability curves

**Figure 1:**
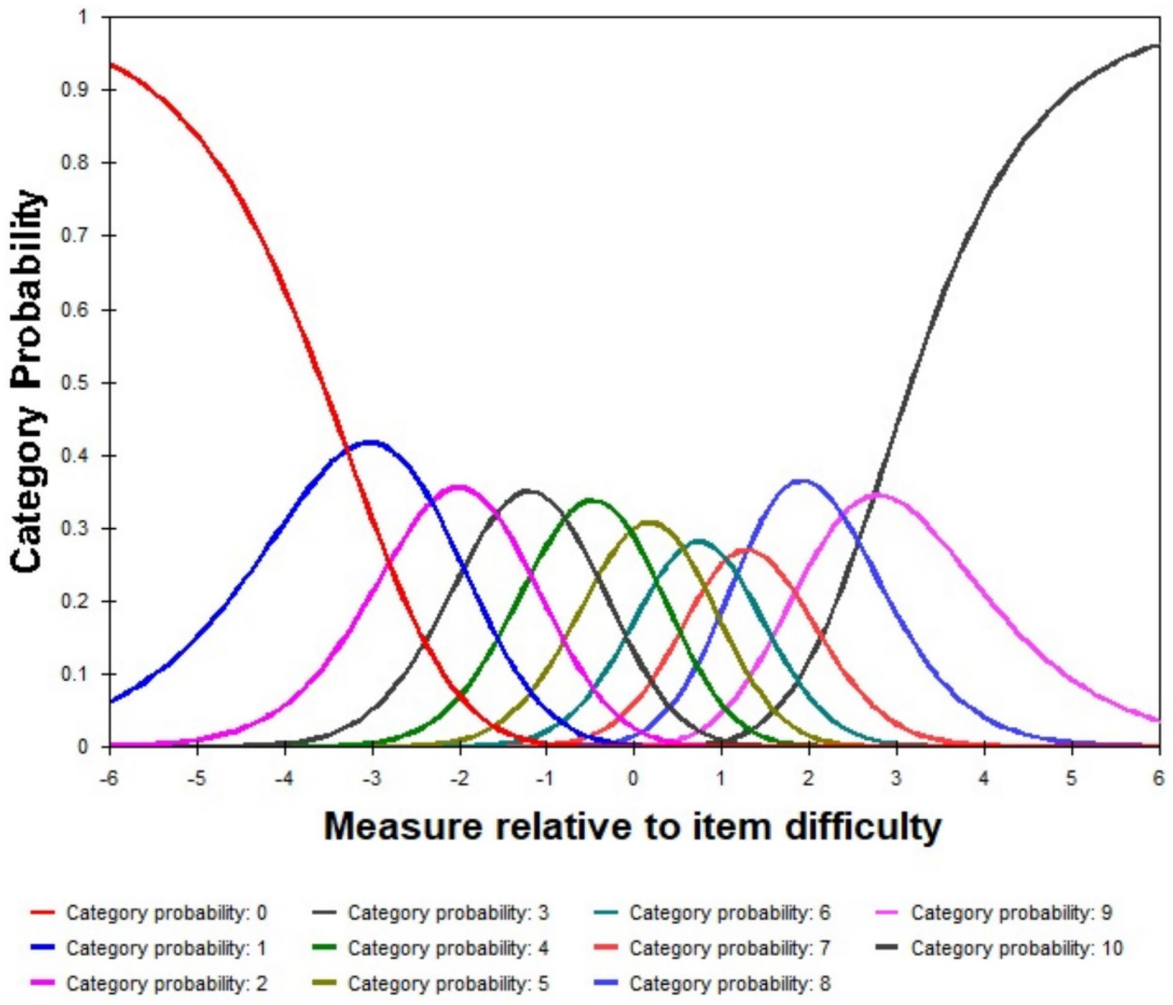
Category probability curves show the probability of selecting each response category at different ability levels (θ) in three dimensions. Each curve represents a specific category, with peaks indicating the most likely response at a given ability level.

Figure 1 presents the category probability curves for the APS-POQ-R-C scale, showing the relationship between item difficulty and individual ability (x-axis) and the response probability for each category (y-axis). The 11 curves, representing rating categories from “0” to “10” indicate whether the peak values are well-separated and consistently ordered. The peaks align with theoretical intersection points, confirming the category options’ effectiveness. The middle categories (“4″,"5″,"6″) were most likely chosen by individuals with intermediate ability, the high-end categories (8–10) was empirically justified by unclear peaks, sparse response frequencies (≤1% per category), disordered threshold intervals (7 → 8: 0.23 logits; 9 → 10: 0.36 logits) violating Linacre’s 0.4–1.0 logit criterion, indicating compromised clinical interpretability (Linacre, 2013), suggesting they may need to be merged. The remaining categories exhibited smooth, continuous curves, effectively distinguishing ability levels and providing valuable information.

### Item characteristics

Table 1 shows the item characteristics. Most items had infit MNSQ and outfit MNSQ indices within a reasonable range, which fit well with the Rasch model. The MNSQ values of item P10 were all greater than 1.50, indicating that the observed distribution of the item deviated significantly from the model’s predicted values, with large fit errors. This may be due to semantic issues with the item itself or individuals’ misunderstanding of the item. Specifically, the content of P10 is “10. Did you receive any information about your pain treatment options? If yes, please circle the number that best shows how helpful the information was:0–10.” This item consists of two sentences, one about obtaining information and the other about the helpfulness of the information. It is speculated that some raters may have mistakenly rated the amount of information obtained instead of the helpfulness of the information. This ambiguous item wording may have led to a mismatch between the observed and expected values, resulting in a loss of measurement accuracy. It is recommended to modify the item wording or delete the item. A reanalysis of the item fit statistics was conducted, the MNSQ values for all remaining items fall within the acceptable range of 0.5 to 1.5, indicating good fit to the model. This suggests that the remaining items show appropriate discrimination and effectively distinguish different levels of pain management outcomes, confirming the stability and validity of the scale without Item P10.

**Table 1:** Item fit and DIF analysis based on age and gender.

Item	Infit MNSQ	Outfit MNSQ	Age DIF contrast (*p*)	Gender DIF contrast (*p*)
P1 least	0.61	0.63	0.27 (0.0089)	0.31 (0.0032)
P2 worst	0.89	0.89	0.14 (0.1334)	0.38 (0.0021)
P3 severe	0.69	0.71	0.10 (0.3003)	0.46 (0.0001)
P4a inbed	0.99	1.00	0.31 (0.0025)	0.36 (0.0005)
P4b outbed	1.21	1.25	0.24 (0.0125)	0.38 (0.0003)
P4c fallsleep	0.97	0.96	0.27 (0.0122)	0.42 (0.0002)
P4d staysleep	1.32	1.31	0.21 (0.0441)	0.40 (0.0001)
P5a anxious	1.32	1.35	−0.37 (0.0323)	−1.11 (0.0000)
P5b depressed	1.29	1.31	−0.42 (0.0140)	−1.05 (0.0000)
P5c frightened	1.29	1.25	−0.37 (0.0026)	0.78 (0.0000)
P5d helpless	0.99	0.99	−0.43 (0.0013)	0.65 (0.0000)
P6a nausea	0.59	0.61	0.11 (0.2851)	0.05 (1.6737)
P6b drowsiness	0.52	0.53	−0.08 (0.4670)	−0.08 (0.4465)
P6c itching	0.94	0.94	−0.02 (0.8436)	0.16 (0.1173)
P6d dizziness	0.94	0.95	0.10 (0.3571)	0.43 (0.0001)
P7 relieve	0.69	0.68	0.17 (0.0804)	0.05 (0.6087)
P8 participate	0.80	0.82	0.14 (0.1702)	−0.03 (0.7662)
P9 satisfied	0.73	0.75	0.15 (0.1519)	−0.00 (1.0000)
P10 helpful	1.90	1.87	−0.14 (0.1601)	−0.21 (0.0345)

### Person-item maps

**Figure 2:**
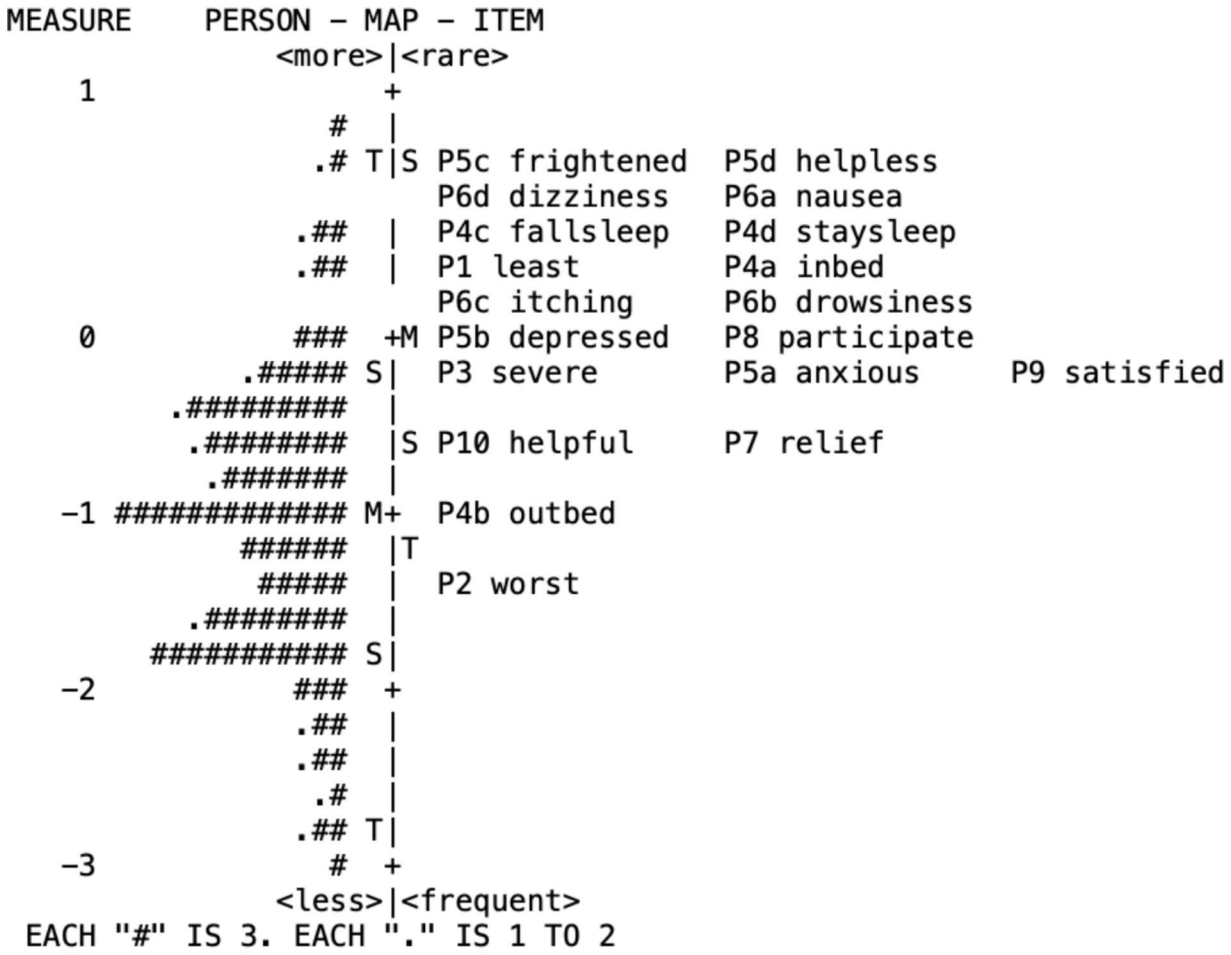
Person-item maps.

Figure 2 displays the person-item map. The mean logit location for the items is 0.0, while the mean logit measure for the individuals is −1.13. This indicates that, overall, the items tend to describe pain conditions or levels that are somewhat more severe than those reported by the majority of individuals in this sample. Consequently, the scale might provide less precise measurement for individuals who report lower levels of pain (those with lower logit measures). Furthermore, the items appear relatively clustered together on the map (i.e., the item distribution is narrow). This suggests potential redundancy or overlap among items, meaning several items might be assessing similar levels of pain experience, potentially contributing limited unique information for differentiating individuals across the spectrum.

### Test information curves

**Figure 3:**
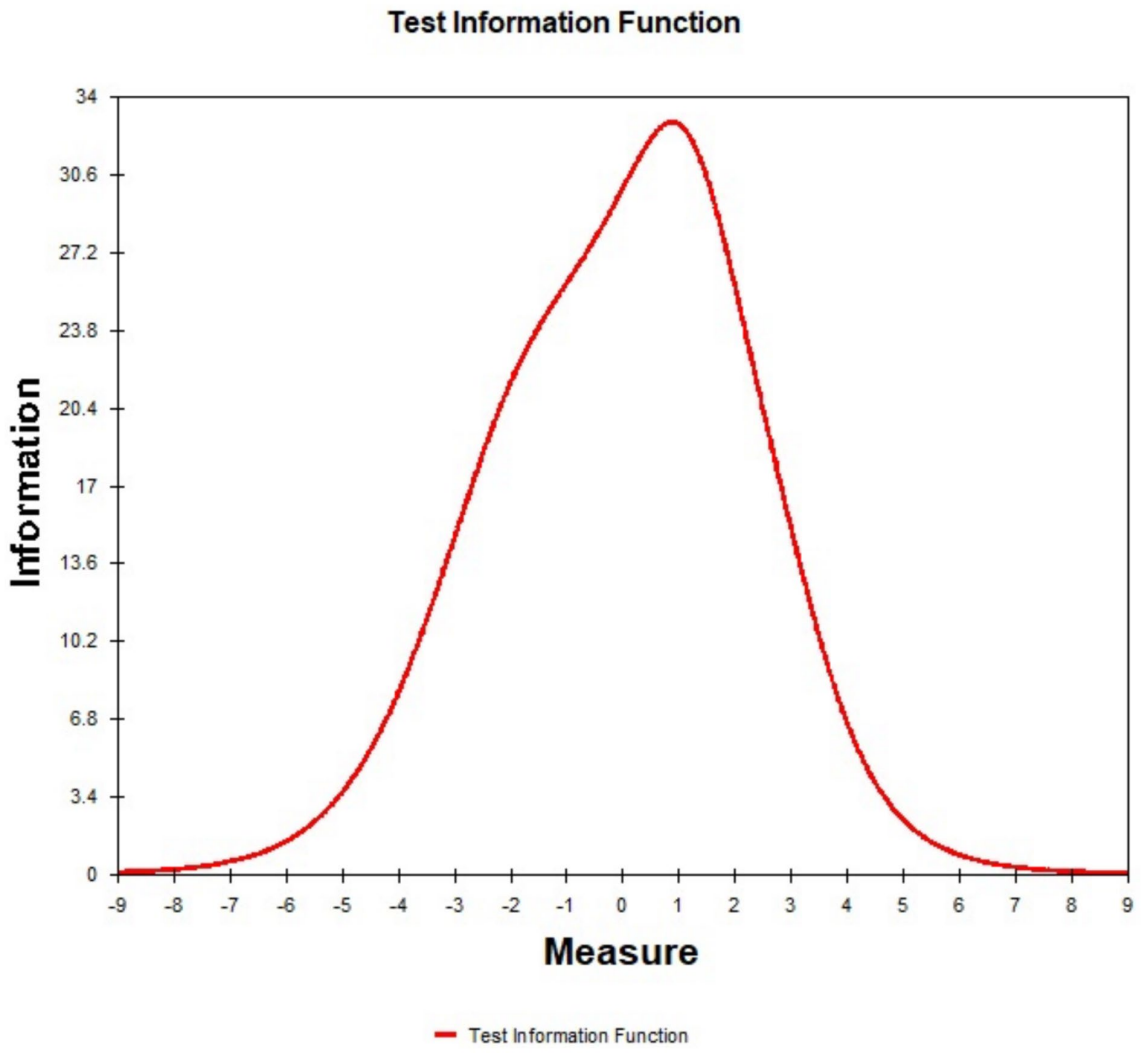
Test information curves represents the measurement precision at different ability levels (θ). The x-axis represents item difficulty versus individual ability, and the y-axis represents information amount.

Figure 3 shows the test information curves, illustrating the scale’s ability to measure individual-item ability levels accurately. The x-axis represents item difficulty versus individual ability, and the y-axis represents information amount. The peak value of each curve indicates the information provided by the items. The larger the peak, the more information obtained, and the wider the peak, the broader the range of applicability. The right-skewed test information function indicates that the scale is more sensitive to individuals experiencing higher levels of pain. This finding aligns with the clinical context of ophthalmic patients, where pain levels are often mild. As a result, the scale may not fully capture the nuances of milder pain, suggesting that its current applicability to ophthalmic patients is limited. To address this limitation, future revisions could include items assessing less severe pain symptoms that better reflect the experience of mild pain, expanding the scale’s utility in broader clinical settings.

### DIF analysis

DIF analysis based on age and gender in Table 1 revealed that, except for the second dimension, most scale items showed no significant DIF. Gender-based DIF was found in items (P5a, P5b, P5c, P5d), with contrast values between greater than 0.5. Specifically, for items P5a and P5b, the female group had significantly higher observed scores than expected, while the male group had lower scores, indicating these items resonated more with females (DIF CONTRAST = −1.11 and −1.05). This may reflect gender differences in anxiety and depression (Chen et al., 2015; Ai and Smyth, 2020). For items P5c and P5d, the female group had lower observed scores than expected, while the male group had higher scores, suggesting these items were more difficult for females (DIF CONTRAST = 0.78 and 0.65), possibly due to gender role stereotypes (Mavridou et al., 2013). To control for Type I error in the gender and age-related DIF analysis, Holm-Bonferroni correction was applied (Holm, 1979). The adjusted *p*-values did not change the results, and the significant DIF findings from the uncorrected analysis remained consistent after correction.

## Discussion

The results of this study show that the APS-POQ-R-C scale has good structural validity. The good unidimensionality, reliability, and separation, ranging from 0.85 to 1.00, indicate that the scale has strong stability and discrimination. Applying this scale in clinical settings can classify patients into different pain management levels, which is crucial for developing differentiated treatment plans and a prerequisite for precise measurement. Most items have good discrimination and fit, indicating that all items can effectively reflect individuals’ latent traits and fit well with the latent response model, with small errors, ensuring the accuracy of measurement. Item P10 have MNSQ values exceeding the threshold, suggesting that these items may be improperly worded or have logical flaws. The person-item map shows that the average item logit location is higher than the average person measure, indicating that most items described situations that fewer patients in this group actually experienced or endorsed, especially those with milder pain. To address this limitation and improve measurement precision at the lower end of the pain spectrum, it is recommended that items assessing less severe pain symptoms be added to the scale. Additionally, repetition and overlap between certain items were observed, indicating information redundancy, and item reduction should be considered. The information function curves suggest that the scale is most applicable to individuals at the higher end of the latent trait spectrum, specifically those with severe pain and poor pain control evaluations. This is consistent with the typical pain levels observed in more severe pain populations and reflects the limited applicability of the scale to populations experiencing mild pain, such as those typically seen after ophthalmic surgery (Lesin et al., 2015). To improve its utility in broader patient populations, it is recommended to add items assessing less severe pain symptoms to better capture the range of pain experiences. The DIF analysis results revealed a slight DIF for some items, mainly in the second dimension of psychological perception, i.e., systematic scoring differences between male and female groups of equal levels on these items. This may be related to cultural factors such as gender roles and emotional expression patterns. For example, society’s expectation of feminine characteristics may influence women’s expression of their own emotions (Anderson et al., 2016). This finding indicates that in relevant tests, we must pay attention to the potential impact of gender differences on symptom self-assessment and be aware of their biasing effect on result interpretation. In future research, it will be necessary to more comprehensively examine the self-description patterns of different groups to improve the fairness and validity of measurement.

Items P1 and P2, which assess mild and severe pain, respectively, can more intuitively reflect individuals’ actual sensations and accurately identify patients at different pain levels. However, we also noticed that the selection frequency of the highest rating category, “10 points,” in item P2 was very low, suggesting that the description of the most severe pain was too extreme for most postoperative ophthalmic patients and did not match reality. Combining the results of the scale’s monotonicity analysis, it is recommended to lower the score setting of the highest rating category to “8 points.” Item P3 assesses the frequency of pain occurrence through the selection of percentages, and the results show that this item has an extremely high degree of fit with the model and can well distinguish different frequency levels of pain. However, the item [P3] itself is located at a higher position in the person–item map, indicating that relatively few patients reported experiencing pain with such high frequency or persistetence pain. This aligns with the observation that this level of pain frequency was not commonly reported by most patients, which also reflects the overall mildness of pain caused by ophthalmic surgery, consistent with clinical experience (Henzler et al., 2004). The positions of items P4a and P4b on the person-item map show that fewer individuals endorsed pain impacting their in-bed activities compared to its impact on out-of-bed activities. This suggests that pain affecting in-bed activity might represent a more severe level of interference for this patient group. For most patients after ophthalmic surgery, basic in-bed activities do not significantly aggravate pain sensations, but pain is more likely to occur during out-of-bed activities and walking, possibly due to the increased stimulation of the incision by the larger range of motion (Brennan, 2011). Items P4c and P4d both assess the impact of pain on sleep, and the results show that these two items [P4c, P4d—sleep impact] are located at higher positions, indicating that most subjects did not endorse their pain significantly impacting sleep. This reflects the limited impact of pain caused by ophthalmic surgery on sleep. We also found that they exhibit a high degree of overlap in the scale structure. The highly similar content of the two items indicates that they contain redundant information. For the above reasons, it is recommended to retain item P4d, which has a greater impact, and remove the redundant item P4c to reduce similar information and improve the simplicity of the structure.

The positions of items P5c-P5d [fear, helplessness] in the person-item map are generally high, indicating that most people did not report feeling these particular negative emotions due to their pain. This suggests that after ophthalmic surgery, the impact of pain on negative emotions is not significant (Coppens et al., 2002). Items P5a and P5b [anxiety, depression] are located at relatively lower positions, indicating that these two emotional responses were more commonly reported by patients in relation to their pain compared to fear and helplessness. This indicates that the occurrence of these extreme negative emotions is very rare after ophthalmic surgery, and most patients can maintain a relatively good and rational state of mind, which is consistent with the overall situation of relatively minor damage and good prognosis of ophthalmic surgery. This also indirectly verifies the rationality of the scale structure, showing that the settings of most items are in line with clinical reality. The actual results of the scale revealed that females are more likely to exhibit certain negative emotional responses, which may be related to physiological factors and social and cultural norms (Gardener et al., 2013). Further research is needed to explore the causes of these differences, which will help us adopt more personalized strategies in subsequent interventions. At the same time, the scores of the female group have systematic deviations from expectations. Among individuals with consistent abilities, females are more likely than males to express anxiety and depression but find it difficult to express negative emotions such as fear and helplessness, which are considered weak. This DIF phenomenon is likely to originate from differences in gender role norms and related psychological repression in the sociocultural background (Anderson et al., 2016). This may not only affect the accuracy of relevant measurements but also suggest that we need to pay attention to the potential impact of gender differences on symptom self-assessment and be aware of their biasing effect on result interpretation. In future research, it will be necessary to more comprehensively examine the self-description patterns of different groups to improve the fairness and validity of measurement. Additionally, the information discrimination range of these four items is relatively narrow, suggesting functional overlap and a limited discrimination effect on individuals. It is recommended to reduce the number of relevant items and retain only one or two items with the greatest information contribution.

Items P6a-P6d assess different types of drug side effects, including nausea, drowsiness, itching, and dizziness. The results show that the positions of the four items in the person–item map are generally high, with item overlap, suggesting that drug side effects caused by pain management after ophthalmic surgery are relatively mild. Among them, dizziness and nausea were slightly more common, indicating a lower frequency of occurrence, while drowsiness and itching were slightly less common, indicating that they were more common. This suggests that dizziness is a more severe reaction with fewer occurrences, while itching and drowsiness are milder common reactions. Items P7-P10 focus on the assessment of pain management satisfaction. The results show that these items are located at higher positions in the scale structure. Considering that the scoring was reversed, this suggests that patients have greater satisfaction and better evaluation of pain control, which is consistent with the overall lower pain perception. Among them, item P10 has a poor fit, most likely due to a certain degree of ambiguity in the wording of the question. Subjects may misinterpret the question requirements. It is recommended to further clarify the meaning of this question to ensure that subjects correctly understand the content the question intends to express or to directly simplify the item statement to reduce possible ambiguity (Linacre, 2002). Additionally, the positions of the pain management evaluation items are relatively concentrated, with overlap and limited discrimination. It is recommended to merge some items and further expand other relevant items on this basis, such as evaluations at the professional level and communication, to obtain more comprehensive feedback from patients on medical services.

Previous studies on this scale have used traditional empirical statistical methods (Gordon et al., 2010; Dihle et al., 2008; Fang et al., 2017; Wang H. et al., 2017) that rely on sample distribution characteristics. However, these methods have certain inherent flaws, such as the inability to compare between samples and the assumption of equal score intervals, which is inconsistent with reality. These two points seriously limit the scientific nature and generalizability of these studies. In contrast, this study introduces the Rasch model from item response theory. This method can effectively solve the above problems by establishing a probabilistic measurement model, calibrating each sample into the same coordinate system, achieving comparability between different samples, converting raw scores into logit values, equalizing the intervals, more realistically reflecting structural differences, and improving the robustness and scientific interpretation of the results. Rasch analysis makes item parameter estimation more accurate and ability measurement more refined, providing a reliable basis for subsequent research.

The study also revealed DIF in the scale among different populations (genders), which may be related to sociocultural differences, such as differences in gender role norms and emotional expression patterns. For example, are females more reluctant to express certain negative emotions that are considered “weaks” (Gardener et al., 2013)? How do these norms influence self-description? We need to conduct in-depth research to explore the specific impact mechanisms of these cultural norms and stereotypes. This not only scientifically guides us to avoid relevant measurement biases but also helps transform and eliminate these potential solidified influences, which has important social significance.

This study also has certain limitations. First, the samples were all from the same hospital and cannot fully represent the entire population. Second, longitudinal follow-up was not conducted, so it was not possible to observe treatment follow-up conditions. Furthermore, large-scale validation is necessary after scale optimization to observe its discrimination ability. Most importantly, the sample population consisted of adult patients who underwent ophthalmic surgery. Due to the minor trauma and mild pain levels of ophthalmic surgery, as well as the relatively limited postoperative emotional impact, the utilization rate of the highest score options in the scale is low, and the item information is more concentrated. This indicates a clear disconnect between the sample’s typical experience and the scale’s focus on items representing higher levels of pain impact. This limits its ability to distinguish patients with mild pain, resulting in many individuals on the left side of the information function curve but limited contribution to the amount of information. This also clearly suggests the relatively narrow applicable range of the scale. Therefore, we must recognize that the advantages of this scale are mainly its ability to distinguish patients with moderate to severe pain, while its ability to distinguish patients with mild pain is relatively limited. This is related to the fact that this study focused only on the specific population receiving ophthalmic surgery. When using the scale, we must consider this limitation and avoid inappropriate generalization.

This study selected the APS-POQ-R-C scale as the research object and applied the Rasch method to test it in postoperative ophthalmic patients. The results show that the APS-POQ-R-C scale has good psychometric properties and a scientific structure and can be used to evaluate postoperative pain in ophthalmic patients effectively, providing support for individualized and precise pain intervention. Of course, the study also revealed the optimization space of the APS-POQ-R-C scale in certain aspects. We provided detailed suggestions for improvement, indicating the direction for the scale’s subsequent development. It is particularly worth mentioning that we paid attention to the important explanatory factor of sociocultural differences, which not only enriched the research perspective but also provided beneficial inspiration for relevant social science research.

## Data Availability

The raw data supporting the conclusions of this article will be made available by the authors, without undue reservation.
