# Supplementary material for: Evaluating pain outcomes in Chinese ophthalmology patients using the APS-POQ-R-C: a Rasch analysis
Source: Front Psychol. 2025 May 5;16:1558111. doi: 10.3389/fpsyg.2025.1558111 (PMC12087573; doi:10.3389/fpsyg.2025.1558111)
Supplement: Supplementary file 2 [file Supplementary_file_2.pdf]

The following questions are about pain you experienced during
the first 24 hours in the hospital <u>or</u> after your operation.

1. On this scale, please indicate the **least** pain you had in the first 24 hours:

0	1	2	3	4	5	6	7	8	9	10
no pain										worst pain possible

2. On this scale, please indicate the **worst** pain you had in the first 24 hours:

0	1	2	3	4	5	6	7	8	9	10
no pain										worst pain possible

3. How often were you in **severe** pain in the first 24 hours? Please circle your best estimate of the percentage of time you experienced severe pain:

0%	10%	20%	30%	40%	50%	60%	70%	80%	90%	100%
Never in severe pain										Always in severe pain

4. Circle the one number below that best describes how much pain **interfered or prevented you from**:

a. Doing activities in bed such as turning, sitting up, repositioning.

0	1	2	3	4	5	6	7	8	9	10
Does not interfere										Completely interferes

b. Doing activities out of bed such as walking, sitting in a chair, standing at the sink.

0	1	2	3	4	5	6	7	8	9	10
Does not interfere										Completely interferes

c. Falling asleep

0	1	2	3	4	5	6	7	8	9	10
Does not interfere										Completely interferes

d. Staying asleep

0	1	2	3	4	5	6	7	8	9	10
Does not interfere										Completely interferes

5. Pain can affect our mood and emotions. On this scale, please circle the one number that best **shows how much the pain caused you to feel**:

	0	1	2	3	4	5	6	7	8	9	10
a. Anxious	0	1	2	3	4	5	6	7	8	9	10
	Not at all										Extremely
b. Depressed	0	1	2	3	4	5	6	7	8	9	10
	Not at all										Extremely
c. Frightened	0	1	2	3	4	5	6	7	8	9	10
	Not at all										Extremely
d. Helpless	0	1	2	3	4	5	6	7	8	9	10
	Not at all										Extremely

6. Have you had any of the following **side effects**? <u>Please circle "0" if no</u>; if yes, please circle the one number that best shows the severity of each:

a. Nausea	0	1	2	3	4	5	6	7	8	9	10
	None										Severe
b. Drowsiness	0	1	2	3	4	5	6	7	8	9	10
	None										Severe
c. Itching	0	1	2	3	4	5	6	7	8	9	10
	None										Severe
d. Dizziness	0	1	2	3	4	5	6	7	8	9	10
	None										Severe

7. In the first 24 hours, how much pan **relief** did you receive? Please circle the one percentage that best shows how much relief you have received from all of your pain treatments combined (medicine and non-medicine treatments):

0% 10% 20% 30% 40% 50% 60% 70% 80% 90% 100%

No Relief Complete Relief

8. Were you **allowed to participate in decisions** about your pain treatment as much as you wanted to?

0 1 2 3 4 5 6 7 8 9 10

Not at all Very much so

9. Circle the one number that best shows how **satisfied** you are with the results of your pain treatment while in the hospital:

0 1 2 3 4 5 6 7 8 9 10

Extremely Dissatisfied Extremely Satisfied

10. Did you receive any **information** about your pain treatment options? ___ No, ___ Yes,

a. If yes, please circle the number that best shows **how helpful** the information was:

0 1 2 3 4 5 6 7 8 9 10

Not at all helpful Extremely helpful

11. Did you use any **non-medicine methods** to relieve your pain? _____ No _____ Yes, if yes, **check all** that apply:

_____ cold pack
_____ deep breathing
_____ distraction (such as watching TV, reading)
_____ heat
_____ imagery or visualization
_____ massage
_____ other (please describe) ________________________________
_____ meditation
_____ listen to music
_____ prayer
_____ relaxation
_____ walking

12. How often did a nurse or doctor **encourage you to use** non-medication methods?

_____ Never _____ Sometimes _____ Often
